# Postpartum Vaginal Adhesion Following Episiotomy: Treatment With Tampon Therapy

**DOI:** 10.7759/cureus.100474

**Published:** 2025-12-31

**Authors:** Nalini Sharma, Isha Purohit, Chelsi Purohit

**Affiliations:** 1 Obstetrics and Gynaecology, Geetanjali Medical College, Udaipur, IND; 2 Obstetrics and Gynaecology, Geetanjali Medical College and Hospital, Udaipur, IND

**Keywords:** conservative medical management, episiotomy complication, postpartum vaginal stenosis, vaginal adhesion, vaginal dilation

## Abstract

Postpartum vaginal agglutination/stenosis is a rare complication. It can lead to significant morbidity related to quality of life and sexual functioning. Documentation in the literature is limited, especially in low-resource settings.

We present a case of complete vaginal stenosis in a 23-year-old primigravida homemaker from Udaipur, India, after a difficult episiotomy repair. The woman presented three months postpartum with dyspareunia and was found to have complete vaginal stenosis approximately 3 cm from the introitus on examination. The patient improved completely with conservative management consisting of sequential vaginal dilation with progressively larger tampons (10-15 mm), with complete resolution within two months and the resumption of intercourse without complication.

This case emphasizes the necessity for timely follow-up of postpartum women to avoid, timely diagnose, and manage complications. The case demonstrates that tampon therapy is a novel, cost-effective, patient-centered, minimally invasive alternative to surgical adhesiolysis in the case of postpartum vaginal stenosis.

## Introduction

Acquired vaginal stenosis, though very rare, is still a menacing problem in underdeveloped countries, linked to the use of chemical vaginal pessaries used as abortifacients, treatment of vaginal discharge, amenorrhea, infertility, etc. [1]. This condition can cause a myriad of symptoms in a patient, including dyspareunia, vaginal pain, dryness, discomfort while using the bathroom, and difficulty with menstrual flow. They not only hamper a woman’s life but also her sexual desires. In this case report, we present a woman who presented to the outpatient department with complaints of dyspareunia and was found to have severe vaginal stenosis secondary to untreated vaginal lacerations resulting from a traumatic episiotomy.

## Case presentation

A 23-year-old primigravida presented to the gynecology OPD with her husband, complaining of inability to resume sexual activity after a vaginal delivery three months ago. She had not resumed her menstrual cycles since delivery. There was no complaint of foul vaginal discharge or spotting. Her labor was prolonged and difficult, and a mediolateral episiotomy was performed to facilitate delivery.

As per the history provided, neither the patient nor her husband followed any specific post-delivery instructions, nor did they attend postpartum follow-up visits. She delivered a healthy 2.5 kg male child at a tertiary care center. The patient reported intense pain and psychological distress due to the traumatic delivery and mentioned feeling embarrassed when discussing her symptoms.

The patient had not menstruated in the three months following delivery. There was no history of chemical irritants in the vagina. On general examination, she was afebrile with a pulse of 80 per minute and blood pressure of 120/70 mmHg. She appeared anxious but was otherwise well. Cardiovascular, respiratory, and abdominal examinations were unremarkable.

Examination of the external genitalia revealed normal labia majora and minora. A healed episiotomy scar was visible. On attempting digital vaginal examination, the vagina was found to be blind-ended. A single finger could not be inserted. Gentle probing revealed dense fibrotic tissue approximately 3 cm from the vaginal introitus; the vaginal cavity was obliterated beyond this point. There was no bulge, adhesion, or significant pain on palpation, only mild discomfort.

Ultrasonography showed no evidence of hematocolpos or pyometra. The uterine cavity appeared patent. The decision was made to perform an examination under anesthesia (EUA) and proceed with further intervention.

Under anesthesia, a 2 mm Hegar dilator was passed through the fibrotic area, confirming intense adhesions. With gentle teasing and sequential dilation by Hegar dilatators, an opening of approximately 1 cm was achieved.

The patient was started on conjugated estrogen vaginal cream (1 g) coated on a tampon, which was inserted and removed after 3-4 hours, then reinserted after 6-8 hours for a similar duration daily.

At the first follow-up visit, seven days later, healing was excellent with no signs of inflammation. The patient was then shifted to a medium-sized tampon, which could be inserted without manipulation. By the second follow-up visit, she progressed to using a large-sized tampon.

Two months after the procedure, the couple reported successful resumption of sexual activity. On examination, normal vaginal caliber was restored, and the patient expressed complete satisfaction with the outcome.

The minimally invasive nature of this intervention resulted in no surgical complications, such as bladder injury or nerve damage. Additionally, the patient did not require prolonged hospital admission.

## Discussion

Acquired vaginal stenosis and vaginal agglutination are rare yet distressing postpartum complications characterized by partial or complete obliteration of the vaginal canal due to inflammation, fibrosis, or hypoestrogenic tissue changes. They often arise following inadequate perineal repair, infection, or delayed wound healing after episiotomy, leading to adhesion formation between opposing vaginal walls. Clinically, patients may present with dyspareunia, inability to resume sexual intercourse, or obstructed menstrual flow, symptoms that carry substantial psychological and relational burden for young mothers. Surgical adhesiolysis has traditionally been considered the mainstay of treatment; however, it carries significant risk in the postpartum setting, including bleeding, bladder or rectal injury, fistula formation, and persistent pain or dyspareunia, especially in scarred or inflamed tissue planes [2]. After an extensive search of the research database, we only found two cases similar to ours; Kim et al reported a case of vaginal birth after cesarean delivery (VBCD), which led to vaginal stenosis secondary to dystocia five months after delivery [3]. In another case, Krimou et al. reported a case of a 19-year-old with a 2-year history of amenorrhea secondary to complete vaginal stenosis caused by unsutured vaginal tears due to labor without medical assistance [4].

In our case, the pathophysiology behind postpartum vaginal stenosis involved an abnormal healing response due to unhealed vaginal lacerations as a result of episiotomy, which led to fibrous tissue formation and eventually vaginal stenosis. The treatment of vaginal stenosis caused by adhesions is primarily surgical. In most cases, adhesiolysis followed by the use of vaginal dilators is preferred due to its simplicity and high success rate. However, reconstructive services have also been used in order to create a neovagina. For example, the pudendal thigh flap, sigmoid vaginoplasty, and McIndoe’s procedure [5-7].

Our case highlights a successful, conservative alternative using tampon-based mechanical dilation combined with topical vaginal estrogen therapy. This approach directly addresses the dual pathophysiology of mechanical adhesion and hormonal insufficiency in the postpartum period. The tampon provides uniform, circumferential pressure along the vaginal walls, enabling gradual expansion rather than abrupt tissue disruption. Its mild hygroscopic swelling creates additional gentle traction, while repeated insertion and removal provide controlled shear forces that gradually break early adhesions. When coated with topical conjugated estrogen, the tampon serves as an effective sustained-release system, maintaining prolonged mucosal contact for 6-8 hours, thereby enhancing epithelial regeneration, vascularization, and elasticity. This synergistic mechanism promotes restoration of normal vaginal patency with minimal discomfort or risk.

Compared with surgical adhesiolysis, our tampon-estrogen therapy offers distinct advantages in terms of safety, cost-effectiveness, and accessibility. The entire course of treatment was inexpensive, required no prolonged anesthesia or hospitalization, and could be largely managed at home, promoting patient autonomy and compliance. Importantly, avoiding surgery eliminates the risk of operative complications and reduces the potential for new scar formation-a key limitation of adhesiolysis, which often necessitates postoperative dilator use to prevent recurrence. Moreover, this approach is particularly valuable in low-resource or high-volume postpartum settings where access to specialized surgical facilities is limited. Its practicality and noninvasive nature make it a viable first-line option for recent-onset stenosis in motivated patients.

Our findings align with existing evidence on the role of local estrogen in improving vaginal mucosal integrity and healing. Topical estrogen enhances epithelial proliferation, collagen deposition, and vascularity, counteracting the hypoestrogenic milieu common in the early postpartum phase. Krimou et al. (2020) reported a case of complete vaginal stenosis requiring surgical correction and long-term dilator use, underscoring the challenges of delayed or inadequate conservative management. In contrast, our case demonstrates that timely intervention with tampon-estrogen therapy can achieve complete resolution without recurrence, obviating the need for surgical reconstruction.

This case underscores the importance of early recognition and intervention in postpartum vaginal stenosis. Conservative management with tampon-based mechanical dilation and topical estrogen is a safe, effective, and patient-centered approach that addresses both mechanical and hormonal contributors to the disease process. With proper patient selection - recent onset, minimal fibrosis, and absence of infection - this method offers an accessible and reproducible option to restore vaginal function while minimizing morbidity.

## Conclusions

Postpartum vaginal stenosis is a rare but significant complication of childbirth, often resulting from abnormal healing of vaginal trauma. In our case, deep episiotomy led to fibrotic tissue formation and stenosis, effectively treated with mechanical adhesiolysis via dilators, followed by topical therapy with estrogen-coated tampons. This highlights the importance of timely postpartum care to prevent such complications and the need for individualized treatment strategies to restore function and improve quality of life.
